# End-point RT-PCR based on a conservation landscape for SARS-COV-2 detection

**DOI:** 10.1038/s41598-022-07756-6

**Published:** 2022-03-19

**Authors:** Armando Cruz-Rangel, Laura Gómez-Romero, Mireya Cisneros-Villanueva, G. de Anda Jáuregui, Victor Luna-Pineda, Alberto Cedro-Tanda, Abraham Campos-Romero, Alfredo Mendoza-Vargas, J. P. Reyes-Grajeda, Alfredo Hidalgo-Miranda, COVID-19 Consortium INMEGEN, Luis A. Herrera, Alfredo Hidalgo-Miranda, Alfredo Mendoza-Vargas, Juan P. Reyes-Grajeda, Felipe Vadillo-Ortega, Alberto Cedro-Tanda, Fernando Peñaloza, Emmanuel Frías-Jimenez, Rosaura Ruiz, Ofelia Angulo, Mireya Cisneros-Villanueva, Jose S. Hernandez-Morales, Bernardo Moreno, Irwin A. Hernández-Cruz, César A. Herrera, Francisco García, Miguel A. González-Woge, Paulina Munguía-Garza, Fernando Luna-Maldonado, Antonia Sanchez-Vizcarra, Vincent G. Osnaya, Nelly Medina-Molotla, Yair Alfaro-Mora, Rodrigo E. Caceres-Gutiérrez, Laura Tolentino-Garcia, José Luis Moreno-Camacho, Jorge Rodriguez-Gallegos, Marco A. Luna-Ruiz Esparza, Miguel Ángel Fernández Rojas, Patricia Rosas-Escobar, Sergio A. Román-González, Marco A. Escobar-Arrazola, Julio C. Canseco-Mendez, Diana R. Ortiz-Soriano, Julieta Dominguez-Ortiz, Ana D. Gonzalez-Barrera, Diana I. Aparicio-Bautista, Griselda Rodriguez Martinez, Armando Cruz Rangel, Luis A. Herrera, Felipe Vadillo-Ortega

**Affiliations:** 1 Biochemistry of Chronic Diseases Laboratory, National Institute of Genomic Medicine, INMEGEN, Mexico City, Mexico; 2 Computational Genomics Department, National Institute of Genomic Medicine, INMEGEN, Mexico City, Mexico; 3 Cancer Genomics Laboratory, National Institute of Genomic Medicine, INMEGEN, Mexico City, Mexico; 4 Cátedras CONACYT Program for Young Researchers, National Council for Science and Technology, CONACYT, México City, México; 5 Research Unit in Immunology and Proteomics, COVID-19 Research Laboratory, Children’s Hospital of Mexico “Federico Gómez”, Mexico City, Mexico; 6 Sequencing Unit, National Institute of Genomic Medicine, INMEGEN, Mexico City, Mexico; 7 National Institute of Genomic Medicine, INMEGEN, Periférico Sur 4809, Arenal Tepepan, Tlalpan, 14610 Mexico City, Mexico; 8 Unidad de Vinculación de la Facultad de Medicina, UNAM en el INMEGEN, Periférico Sur 4809, Arenal Tepepan, Tlalpan, 14610 Mexico City, Mexico; 9 Center for Complexity Sciences (C3), National Autonomous University of Mexico, Mexico City, Mexico; 10 Biomedical Research Unit in Cancer, National Institute of Cancerology, Mexico City, Mexico; 11 Institute of Biomedical Research, National Autonomous University of Mexico, Mexico City, Mexico; 12 Innovation and Research Department, Salud Digna, Culiacan, Sinaloa Mexico; 13 Secretary of education, science, technology and innovation, Mexico City, Mexico

**Keywords:** Computational biology and bioinformatics, Molecular biology, Biomarkers

## Abstract

End-point RT-PCR is a suitable alternative diagnostic technique since it is cheaper than RT-qPCR tests and can be implemented on a massive scale in low- and middle-income countries. In this work, a bioinformatic approach to guide the design of PCR primers was developed, and an alternative diagnostic test based on end-point PCR was designed. End-point PCR primers were designed through conservation analysis based on kmer frequency in SARS-CoV-2 and human respiratory pathogen genomes. Highly conserved regions were identified for primer design, and the resulting PCR primers were used to amplify 871 nasopharyngeal human samples with a previous RT-qPCR based SARS-CoV-2 diagnosis. The diagnostic test showed high accuracy in identifying SARS-CoV-2-positive samples including B.1.1.7, P.1, B.1.427/B.1.429 and B.1.617.2/ AY samples with a detection limit of 7.2 viral copies/µL. In addition, this test could discern SARS-CoV-2 infection from other viral infections with COVID-19-like symptomatology. The designed end-point PCR diagnostic test to detect SARS-CoV-2 is a suitable alternative to RT-qPCR. Since the proposed bioinformatic approach can be easily applied in thousands of viral genomes and over highly divergent strains, it can be used as a PCR design tool as new SARS-CoV-2 variants emerge. Therefore, this end-point PCR test could be employed in epidemiological surveillance to detect new SARS-CoV-2 variants as they emerge and propagate.

## Introduction

*Severe acute respiratory syndrome coronavirus 2* (SARS-CoV-2) is the etiological agent causing the severe respiratory disease coronavirus disease 2019 (COVID-19) global pandemic. SARS-CoV-2 emerged in 2019, having its epicenter in an animal market in Wuhan Province, Hubei, China^1^. To date, it has infected more than 440 million people and caused over 5.5 million deaths^2^.

The rapid detection of SARS-CoV-2 is crucial to mitigate COVID-19 propagation. Therefore, diagnostic tests are and will continue to be essential in the containment and epidemiological surveillance of SARS-CoV-2. Two main methodologies have been developed for SARS-CoV-2 detection. The first one, also known as the “molecular test”, is based on viral RNA extraction from nasopharyngeal swabs, and involves amplification of specific sequences of the SARS-CoV-2 genome using the *reverse transcription-polymerase chain reaction* (RT-PCR) technique, either the gold standard *real-time quantitative reverse transcription PCR* (RT-qPCR) or end-point PCR^3^. RT-qPCR is a highly sensitive, easy-to-implement and rapid technique; however, its high-throughput application poses an economic burden for developing countries, in which it has been a challenge to keep up with the demand for detection tests. The second methodology, the “immunologic test”, consists of probing serum from patients for either specific antibodies against SARS-CoV-2 or probing nasopharyngeal swab samples for specific viral proteins^4,5^. These methodologies (molecular and immunological) are useful in different stages of the course of COVID-19, i.e., tests based on the identification of viral genome fragments allow diagnosis during the acute phase of the infection, whereas immunological tests identify individuals who have already developed response antibodies to the virus^6^.

Moreover, the emergence of new SARS-CoV-2 variants with nonsynonymous substitutions in key proteins involved in pathogenesis and virulence processes has allowed the virus to continue along its evolutionary and adaptive process and displace the original Wuhan strain^7–9^, representing a major public health concern. Accordingly, the emergent variants B.1.1.7 from the UK^10,11^, B.1.351 from South Africa^12^, P.1 from Brazil^13,14^ and B.1.617.2/ AY from India have shown higher transmissibility as well as decreased neutralizing activity in monoclonal antibody treatments (isolated either from plasma of COVID-19 convalescent or vaccinated individuals)^15^. Therefore, although some vaccines have already been approved for emergency use, the time required for population immunization and the dispersion of more virulent variants necessitate the development and implementation of efficient low-cost diagnostic tests that require little infrastructure to continue with the epidemiological surveillance of the vulnerable population and allow the containment of COVID-19 outbreaks.

The wide and generalized use of high-throughput sequencing (HTS) has allowed the generation and sharing of enormous numbers of SARS-CoV-2 genomes in a short time, enabling the use of genomic data for faster and better oligo design and epidemiological surveillance.

Highly specific and sensitive PCR detection will require PCR primers capable of amplifying any SARS-CoV-2 genome without any nonspecific products from other bacteria or viruses. Current approaches commonly used for pathogen identification are aimed either 1) to guide PCR design or 2) to characterize existing variation in known primer and probe regions. The first approach presents difficulties as they either rely on complete conservation across the target sequences or do not directly address how natural viral variability could impact the method’s performance; on the other hand, the second approach is not designed to pinpoint regions for new primer generation.

In this work, a methodological approach to guide the design of PCR primers that is tolerant to the SARS-CoV-2 genome variability and does not impose any conservation threshold across all population samples is proposed. Since this approach analyzes genome-wide conservation without focusing on a specific region, it is suitable for use as a discovery tool to identify new regions that could be further analyzed for oligo identification. Based on this approach, several PCR primers were designed to be experimentally tested. A accurate end-point RT-PCR diagnostic test was developed, and its performance was evaluated in 871 clinical samples with a previous RT-qPCR SARS-CoV-2 diagnosis including B.1.1.7, P.1, B.1.427/B.1.429 and B.1.617.2/ AY variants samples. Notably, our diagnostic test showed a positive likelihood ratio greater than 10 and a detection limit of 7.2 viral copies/µL, comparable to the detection limit of RT-qPCR. In addition, we demonstrated that our test can discriminate SARS-CoV-2 from other pathological agents that cause similar symptoms. Finally, this bioinformatic approach could be used as an epidemiological surveillance tool to monitor conservation across regions targeted by existing RT-qPCR assays and to guide the design of new PCR primers as new SARS-CoV-2 variants emerge.

## Material and methods

### Data collection

The SARS-CoV-2 reference genome sequence was downloaded from NCBI (accession: MN908947.3). Bacteria and viruses that can be commonly found in the human respiratory tract were obtained from previous studies^16–18^. All available sequences from these viruses and bacteria were downloaded from the European Nucleotide Archive (ENA). The names of all studied organisms and the number of available sequences per organism are shown in Supplementary Table 1. In addition, genomic sequences from other human coronavirus strains (human coronavirus 229E, human coronavirus OC43 and human coronavirus NL63) were downloaded from ENA (accessions AF304460.1, AY585228.1 and AY567487.2). A total of 1,910 environmental sequences were included in the analysis. SARS-CoV-2 genomic sequences from population samples were downloaded from the GISAID database (GISAID, https://www.gisaid.org, last accessed 05/08/2021). Only genomes labeled in GISAID as “complete and high coverage” were downloaded. Approximately 1000 SARS-CoV-2 genomic sequences were downloaded for each nucleotide substitution or known lineage reported in the database. A total of 35,858 sequences were analyzed. All accessions can be found at Supplementary File 1. The name of each nucleotide substitution or lineage and the number of downloaded sequences are listed in Supplementary Table 2.

### Kmer frequency analysis and identification of candidate primer sequences

For this analysis, a genome of length N is considered a string of length N, and any genomic substring of size k is called a kmer. A kmer obtained from the SARS-CoV-2 reference genome is called a reference kmer.

Three different sets of target genomes were analyzed: i) environmental sequences that could interfere with the specific amplification of SARS-CoV-2, i.e., sequences belonging to viruses and bacteria that can be commonly found in the human respiratory tract (environmental sequences) (Supplementary Table 1); ii) SARS-CoV-2 complete genomic sequences isolated from population samples (SARS-CoV-2 population sequences); and iii) SARS-CoV-2 complete genomic sequences specific for each SARS-CoV-2 lineage (lineage-specific SARS-CoV-2 genomic sequences) (Supplementary Table 2). Lineage-specific genomes were also included in group ii and were further divided into subgroups containing the genomes for each lineage.

All target genomes were subdivided into kmers of length 19 to 31 with a sliding window of 1. For target genome X and kmer size 19: kmer 1 contained nucleotides from position 1 to position 19, kmer 2 contained nucleotides from position 2 to position 20, and so on. For each group (environmental sequences, SARS-CoV-2 population genomic sequences, or lineage-specific SARS-CoV-2 genomic sequences), the frequency of all observed kmers was calculated using Jellyfish v1.1.12^19^. In this step, a Jellyfish database storing kmer frequencies is generated for each combination of kmer size and group (or subgroup) of target genomes.

The script kmer-cov-plot developed by the AMOS consortium^20^ was used to obtain the number of occurrences of each reference kmer in each group (or subgroup) of target genomes. Briefly, kmer-cov-plot splits the SARS-CoV-2 reference genome into reference kmers and looks for each reference kmer in a given Jellyfish database.

Kmers not present in environmental samples and highly conserved among SARS-CoV-2 population samples (present in more than 99% of the genomes) were identified by manual inspection and were chosen as candidate primer sequences since a PCR primer of size k can be considered a kmer. To calculate the physicochemical properties (melting temperature, potential DNA secondary structures or potential primer-dimer formation) of the chosen sequences, the PrimerDimer module from PrimerSuite software was used^21^.

In addition, the specificity of the amplification generated from the chosen sequences was evaluated using the human genome as a background sequence with the online software Primer-BLAST^22^.

### Samples evaluation by end-point RT-PCR

The study was approved by the ethics and research committee of INMEGEN (CI/2/2020/I). All experiments were performed in accordance with relevant guidelines and regulations. Informed consent was obtained from all participants. A total of 871 human samples from nasopharyngeal swabs and sputum with a previous positive RT-qPCR diagnostic were evaluated including 762 random samples and 109 samples that had been previously sequenced by the COVID-19 Consortium INMEGEN. The samples for this study were randomly selected from batches of samples arriving to INMEGEN, obtained from symptomatic patients in multiple public COVID-19 centers and hospitals in Mexico City. Sequencing was done with the COVID-Seq test kit (Illumina, 20,043,675) following manufacturer instructions. The resulting sequences were deposited at GISAID (Supplementary Table 3) and lineage assignment was done using the Pangolin software **(**https://cov-lineages.org/pangolin_tutorial.html**,** last accessed 05/28/2021). RNA extraction was carried out with automated equipment (KingFisher Flex 711–349, Thermo Fisher Scientific) and the MagMax Viral and Pathogen Nucleic Acid Isolation kit (Thermo Fisher Scientific). All samples used in the study were evaluated by RT-qPCR with US CDC real-time RT-qPCR primer/probe sets for 2019-nCoV_N1 and 2019-nCoV_N2 and human RNase P (RP) for SARS-CoV-2 detection before end-point PCR analysis was performed.

For end-point PCR, a reverse transcription master mix (RT buffer, dNTPs, random primers and transcriptase reverse enzyme) was prepared following the manufacturer’s specifications. For each sample, (5–50 ng) of RNA was mixed with 10 µl of master mix, and the resulting mix was subjected to serial incubations in a GeneAmp PCR System 9700 thermal cycler (Applied Biosystems) at 25 °C for 10 min, 37 °C for 120 min, and 85 °C for 5 min and then maintained at 4 °C. Then, 4 µl of the resulting cDNA was mixed with 16 µl of PCR master mix (MasterMix AMPLIQON 2X; forward and reverse specific primers). Primer sequences are listed in Table 2. N1 and RP primers correspond to the primers included in the US CDC real-time RT-qPCR detection protocol. S, E and NSP-3 primers were selected based on sequence conservation, physicochemical properties and specificity of the amplification. The PCR program was optimized to 95 °C for 2 min; followed by 35 cycles of 95 °C for 30 s, 55 °C for 3 s; and 72 °C for 15 s and then maintenance at 4 °C. PCR products were visualized through electrophoresis in 4% agarose gels stained with SYBR Gold (Thermo Fisher Scientific).

**Table 1:** Test performance. A total of 762 samples were tested by end-point PCR to determine the presence of SARS-CoV-2. The samples were previously tested by the gold standard RT-qPCR. 217 samples were used for test NSP3, and 545 samples were used for test E, respectively. 4 and 10 samples, respectively, were diagnosed as inconclusive and excluded from the analysis.

Parameter	Test NSP3 (RP, N1, NSP3, S)	Test E (RP, N1, S, E)
TP	43	139
TN	139	340
FP	1	10
FN	30	28
Sensitivity	0.589 (CI 95%: 0.476,0.701)	0.851 (CI 95%: 0.797,0.906)
Specificity	0.992 (CI 95%: 0.978,1.029)	0.973 (CI 95%: 0.957,0.989)
LR +	82 (CI 95% = (12,587)	32.65 (CI 95%:17,58)
LR-	0.42 (CI 95% = (0.31,0.54)	0.15 (CI 95%: 0.10,0.22)
PPV	0.977 (CI 95%: 0.933,1.021)	0.932 (CI95%: 0.892,0.973)
NPV	0.82 (CI 95%: 0.811,0.833)	0.939 (CI95%:0.924,0.954)
Cohen’s Kappa	0.64 (p.value 9e-31)	0.83 (p.value 3e-208)
N	21,720	545

**Table 2:** PCR fragments to monitor COVID-19.

Name	Sequence	Size (pb)
Nucleoprotein (N1) F (CDC)	5'GACCCCAAAATCAGCGAAAT3'	
Nnucleoprotein (N1) R (CDC)	5'TCTGGTTACTGCCAGTTGAATCTG3'	72
Spike (S) F	5'ACCAGATCCATCAAAACCAAGC3'	
Spike (S) R	5'TGTTTGATGAAGCCAGCATCTG3'	90
Envelope (E) F	5'CTCATTCGTTTCGGAAGAGACAGGTACGTTA3'	
Envelope (E) R	5'TTTTAACACGAGAGTAAACGTAAAAAGAAGG3'	185
NSP-3 F	5'GGCTGTAGTTGTGATCAACTC3'	
NSP-3 R	5'TAAGACGGGCTGCACTTACAC3'	96
RNAse P (RP) F (CDC)	5'AGATTTGGACCTGCGAGCG3'	
RNAse P (RP) R (CDC)	5'GAGCGGCTGTCTCCACAAGT3'	65

A positive was defined as a sample with a positive diagnostic by endpoint RT-PCR, a positive sample could have either a positive or negative diagnostic by RT-qPCR and it was classified as a True Positive (TP) or False Positive (FP), respectively. A negative was defined as a sample with a negative diagnostic by endpoint RT-PCR, a negative sample could have either a positive or negative diagnostic by RT-qPCR and it was classified as a True Negative (TN) or False Negative (FN), respectively. To obtain test accuracy measures, the contingency values of Table 2 were obtained based on the next equations:

Sensitivity = TP/(TP + FN); Specificity = TN/(TN + FP); PPV = TP/(TP + FP); NPV = TN/(TN + FN); LR +  = Sensitivity/ 1 − Specificity; LR- = 1 − Sensitivity/ Specificity and Cohen’s kappa = P0 − Pe/1 − Pe where; PPV (Positive Predictive Value); NPV (Negative Predictive Value); P0 = Overall accuracy of the model; Pe = agreement between the model predictions and the actual class values.

### Standard curve and viral load determination

Copies of the SARS-CoV-2 virus were quantified using a standard curve with serial dilutions using the 2019-nCoV_N and Hs_RPP30 positive controls synthesized by Integrated DNA Technologies (IDT, Coralville, IA). Ct values obtained from each dilution were used to calculate the linear regression values to interpolate the Ct of each gene from the samples included in this study.

A pool of 500 µl of total RNA from patients with negative RT-qPCR results was prepared. Then, serial dilutions with RNA from patients with positive RT-qPCR results (Ct = 16–30) were prepared and mixed with the pool previously described. The results from every serial dilution were monitored and visualized on 4% agarose gels (Fig. 3). The rough estimates of viral load from the diluted samples and those evaluated by end-point PCR were calculated by using the values obtained in the qPCR dilution curves for the N marker.

### Test specificity validation

End-point PCR with the RP, N1, S and E primers was performed using RNA from HcoV-OC43 and H1N1 influenza. In addition, end-point PCR using infA primers (CDC,https://www.who.int/csr/resources/publications/swineflu/CDCRealtimeRTPCR_SwineH1Assay-2009_20090430.pdf, last accessed 05/08/2021) was performed as a positive control for H1N1, whereas primers specific for a fragment of the N gene were used as a positive control for OC43^23^. Viral genetic material was provided by the COVID-19 research laboratory of the Hospital Infantil de México “Federico Gómez” from NATrol Respiratory Verification Panel 2. The primer sequences are shown in Supplementary Table 4.

## Results

### The SARS-CoV-2 conservation landscape can pinpoint regions suitable for oligo design

All available sequences from common bacteria and viruses usually found in the human respiratory tract and three known human coronavirus strains (229E, OC43 and NL63) were analyzed as environmental sequences. We also analyzed 35,858 high-coverage complete SARS-CoV-2 genomic sequences isolated from population samples as SARS-CoV-2 population sequences. A total of 1000 sequences per lineage and each reported mutation in the Global Initiative on Sharing All Influenza Data (GISAID) repository were analyzed.

**Figure 1:**
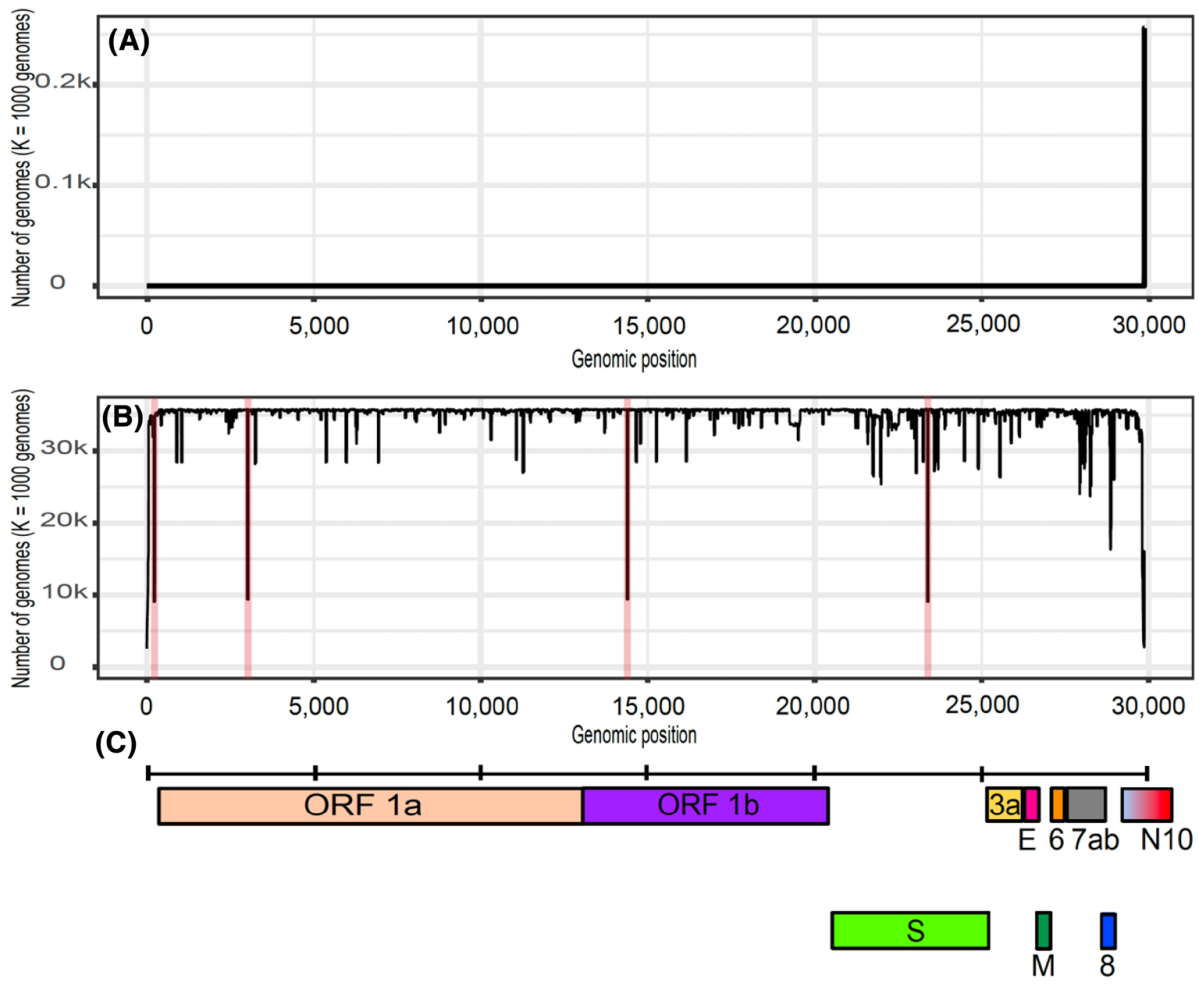
(**A**) Genome-wide environmental conservation landscape. (**B**) Genome-wide population conservation landscape; positions 241, 3037, 14,408 and 23,403 are highlighted in red. In both panels, the X axis represents the genome position and the Y axis represents the number of genomes that contain each reference kmer. (**C**) Genome annotation.

A conservation landscape is the visual representation of the frequency of each possible SARS-CoV-2 reference kmer (with no mutations) along each set of target genomes, i.e., either environmental sequences or SARS-CoV-2 population sequences. We generated SARS-CoV-2 environmental and population conservation landscapes for a wide range of kmer sizes (from k = 19 to k = 31). The SARS-CoV-2 environmental and population conservation landscapes are plotted in Fig. 1. The X-axis represents each reference kmer along the SARS-CoV-2 reference genome, and the Y-axis represents the number of occurrences across either the environmental sequences (Fig. 1A) or the population sequences (Fig. 1B) for each reference kmer. Notably, most reference SARS-CoV-2 kmers have very low frequency values at Fig. 1A which implies that they are not present in any environmental sequence. By other hand, reference SARS-CoV-2 kmers tend to have very high frequency values at Fig. 1B indicating that those reference kmers are present in most of the SARS-CoV-2 population sequences. However, high variation in the frequency values at Fig. 1B are observed suggesting that SARS-CoV-2 genome is highly variable at the population level. Positions 3037, 14,408 and 23,403 show abrupt peaks indicating the presence of the reference allele in a low fraction of SARS-CoV-2 genomes (around 9000 genomes) and the presence of a mutant allele in a high fraction of SARS-CoV-2 genomes (around 21,000 genomes). These mutations correspond to the 3,037C > T silent mutation, the 14,408C > T mutation resulting in RNA-dependent RNA polymerase (RdRp) P323L mutation and the 23,403A > G mutation resulting in the Spike protein D614G. RdRp-P323L and Spike-D614G have been associated with severity of COVID-19. Besides, Spike-D614G increases virion spike density and infectivity^45,46^. Mutation at position 241 is located in the 5’-untranslated region of the virus genome and it was found to be the distinguishing mutation between the two major locally transmitted outbreaks in China, the first one identified in December 2019 in Wuhan and the second one in June 2020 in Beijing-Xinfadi^47^.

**Figure 2:**
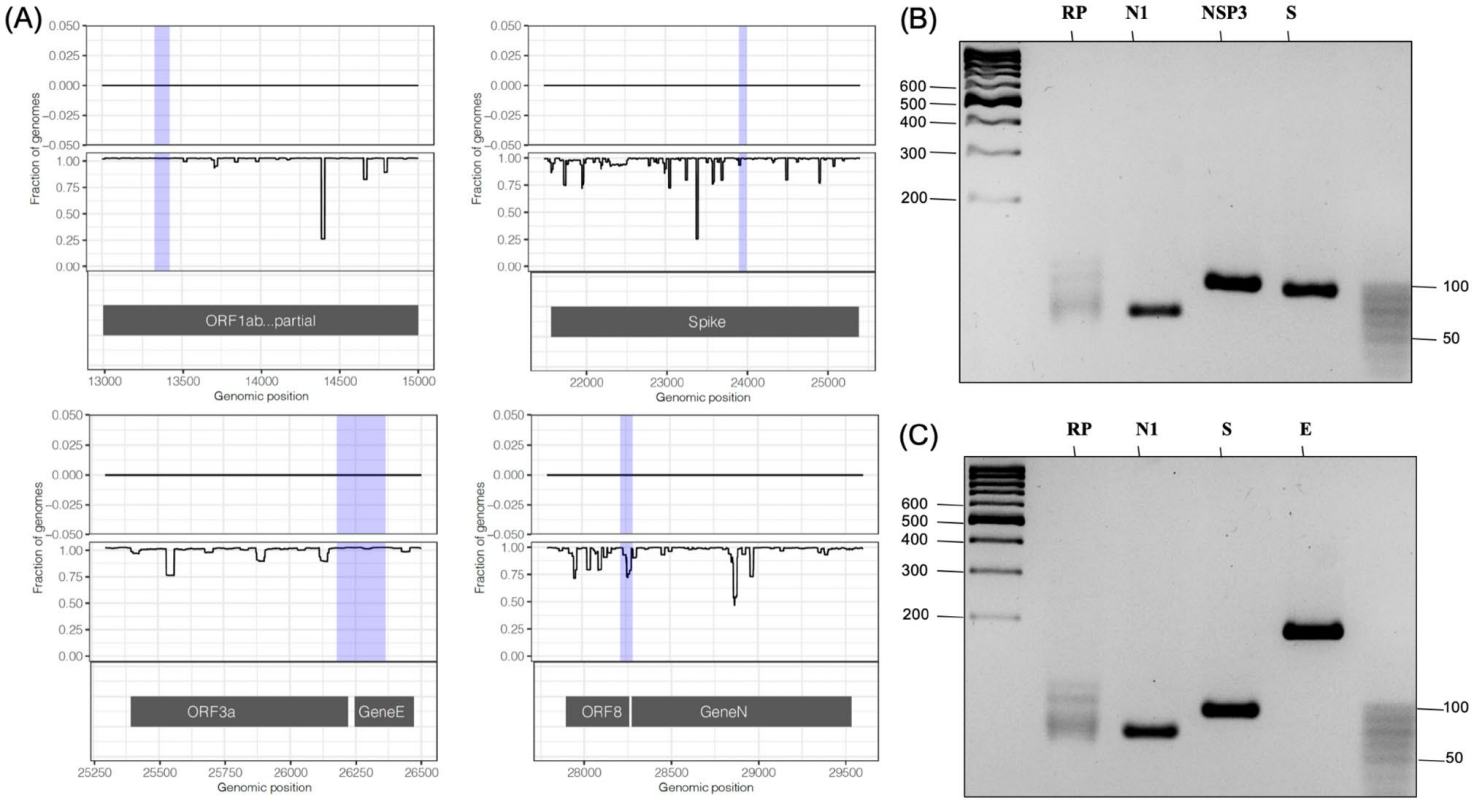
(**A**) Environmental and population landscapes are shown for each selected region. A panel is included for all primers used in this study. The shadowed blue regions represent the amplicons generated by the PCR primers: upper left, primer NSP-3; upper right, primer S; lower left, primer E and lower right, primer N1. *Upper track*: number of occurrences of each reference kmer in the environmental sequences; upper and middle track: X-axis shows the genomic position for the start of each reference kmer, Y-axis, shows the percentage of either environmental sequences (upper track) or SARS-CoV-2 population genomes (middle track) that contain a given reference kmer; *lower track,* genomic annotation. (**B**) 4% agarose gels showing the end-point RT-PCR amplification product when using as sample a positive control (SARS-CoV-2 RNA) with the primers RP and N1 (CDC recommended primers) as well as the NSP3 and S primers generated by the kmer method. (**C**) 4% agarose gels showing the end-point RT-PCR amplification product when using as sample a positive control with the RP, N1, S and E primers.

A region suitable for oligo design will present zero occurrences of the SARS-CoV-2 reference kmers in the environmental conservation landscape (high specificity) and will present N occurrences of the SARS-CoV-2 reference kmers in the population conservation landscape, where N equals the number of population genomes being analyzed (high sensitivity). Thus, the environmental and population conservation landscapes were inspected to select regions suitable for primer generation. Figure 2A shows the environmental and conservation landscape for the selected regions: genomic range 13,000 to 15,000 (primer NSP-3, kmer size = 21); genomic range 21,500 to 25,400 (primer S, kmer size = 22); genomic range 25,300 to 26,500 (primer E, kmer size = 31) and genomic range 27,800 to 29,600 (primer N1, kmer size = 20). The shadowed blue regions represent the amplicons generated by the PCR primers used in this study.

Figure 2A shows the environmental and population conservation landscape for the selected regions. The amplicons generated by the analyzed primers are shown as a shadowed blue region. If a pair of primers is specific for SARS-CoV-2 amplification, no exact occurrences of the kmers surrounding the amplicons in the environmental landscape are expected. In this landscape, a vertical line occurs when a reference kmer is present in at least one environmental sequence, and its height is equal to the number of occurrences of that specific kmer. Notably, in these regions, there is no single reference SARS-CoV-2 kmer in any environmental sequence.

In contrast, a conserved region present in all population genomes will appear as a steady horizontal line close to the total number of samples in the SARS-CoV-2 population landscape. A highly sensitive pair of primers must show high conservation at the kmer corresponding to the amplification primers (borders of the amplicon). A mutation present in all the population genomes will produce a decrease in the number of genomes carrying the reference kmers that overlap the mutation. The sequences of both NSP-3 primers, both E primers, the reverse S primer and the forward N1 primer were present in more than 99% of the SARS-CoV-2 genomic sequences. The forward S primer was present in 93.4% of all population samples, and the reverse N1 primer was found in only 75.81% of the SARS-CoV-2 population genomes (Fig. 2A). Overall, these data suggest highly specific and sensitive SARS-CoV-2 amplification by all the primers analyzed, although the N1 primers could have lower sensitivity.

### End-point RT-PCR tests show high accuracy in determining SARS-CoV-2-positive samples

To design the specific primers to be used for the PCR test, candidate sequences from the bioinformatic analysis for nsp3, S and E were used, along with the CDC primers for the N gene and the endogenous RP control. A total of 765 random samples with a previous RT-qPCR validated diagnosis^24^ were evaluated in this study. A total of 217 samples were used to evaluate the nsp3 and S primers, and the N1 and RP primers were used as controls. By using these primers, 23% of the samples showed a positive result when analyzed by end-point RT-PCR, although 34% were detected as positive by RT-qPCR (Fig. 2B). However, in samples with Ct values higher than 25, nonspecific bands, likely corresponding to the cell genomic bulk, were observed for the nsp3 primer. In contrast, substitution of nsp3 primers by E primers improved the accuracy of the test in an analysis of 545 samples, as 27% of the samples showed a positive band pattern (Fig. 2C) for a positive sample when analyzed by end-point RT-PCR, whereas 30.4% of the samples had a positive result from RT-qPCR. Thus, by using the E primer, the accuracy and precision of the test were improved, as the diagnosis agreed with that reported by RT-qPCR even in samples whose Ct was higher than 34. Samples with indeterminate band patterns were discarded from the statistical analysis. The classification criteria summary is shown in Supplementary Table 5.

The sensitivity and specificity values for the tests were 0.58 and 0.99 for test NSP3 (RP, N1, S, NSP3) and 0.86 and 0.97 for test E (RP, N1, S, E), respectively (Table 1). Although both tests allowed the correct identification of negative cases, test E showed higher sensitivity for detecting positive cases. Regarding test performance, the likelihood ratio positive (LR +) was superior to 10 in both cases, indicating high accuracy for the detection of SARS-CoV-2-positive cases. However, when analyzing test performance in identifying negative cases, the likelihood ratio negative (LR−) value was 0.42 and 0.15 for test NSP3 and test E, respectively, suggesting that test E performs better for identifying SARS-CoV-2-negative cases. The Cohen kappa index for the test NSP3 was 0.64 (good agreement), whereas for the test E it was 0.83, indicating an almost perfect agreement when comparing to the gold standard RT-qPCR.

### Detection limit and viral load

Since test E showed higher sensitivity and specificity in determining either SARS-CoV-2-positive or SARS-CoV-2-negative cases, we chose this test as the diagnostic test. We calculated the number of True Positives and False Negatives for increasing ranges of Ct values. Notably, test E showed an accurate prognosis in samples whose Ct values for the N1 marker were in the range of 15–34, although the diagnosis was less accurate in samples with Ct values greater than 34 since most False Negatives are found at high Ct values (CT > 35) (Figure sup 1).

**Figure 3:**
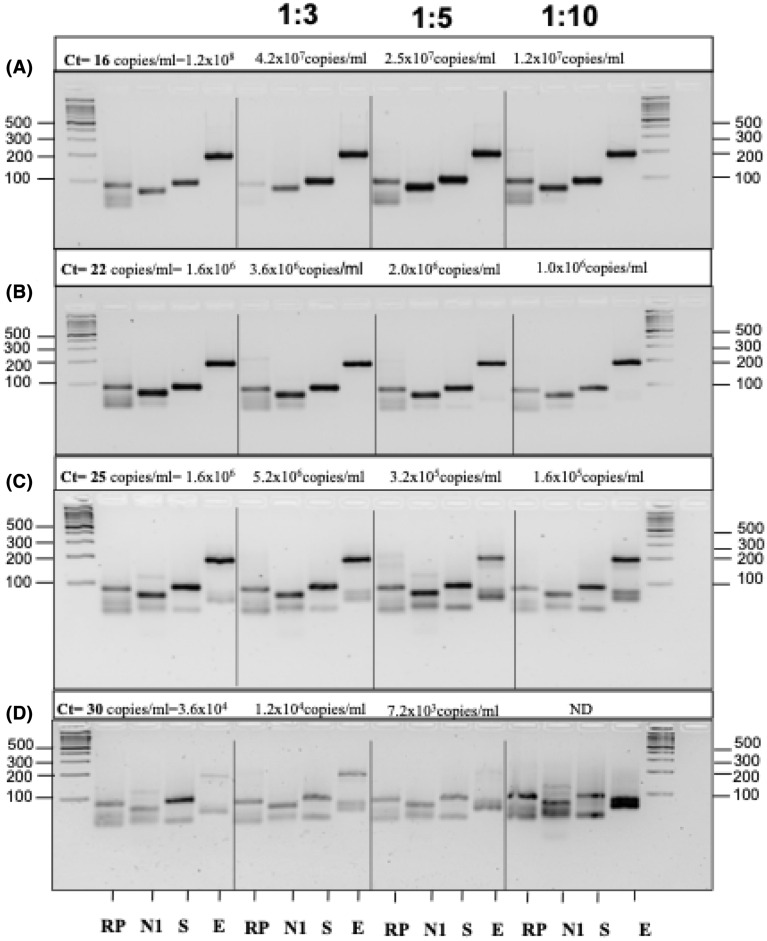
End-point PCR showed a high detection limit when analyzing samples with a positive qRT-PCR diagnosis. All samples in the study had known Ct values that were used to determine viral copy numbers. (**A**–**D**) Increasing Ct values in samples were correlated with lower viral loads. (**D**) The positive sample with a Ct value of 30 corresponded to a detection limit of 7 viral copies/µl (1:5 dilution). At lower dilutions, the virus fragments were no longer detectable, and nonspecific bands of human genomic bulk were observed.

Then, we determined the detection limit of the test by considering that the viral load (number of viral particles/mL) is proportional to the Ct value in a positive sample. For this, serial dilutions from a positive control whose corresponding viral load was provided by the manufacturer (see materials and methods section) were made to determine the Ct value in each serial dilution (Figure sup 2). Thus, an approximate viral load in positive samples with Ct values of 16–30 could be calculated. Our test possessed a detection limit of 7.2–10 viral particles/µL, as shown by the end-point PCR evaluation of serial dilutions from positive samples with a known viral load (Fig. 3).

### Selected primers can be used to detect recently discovered SARS-CoV-2 lineages, and the SARS-CoV-2 conservation landscape can be used as an epidemiological surveillance tool

**Figure 4:**
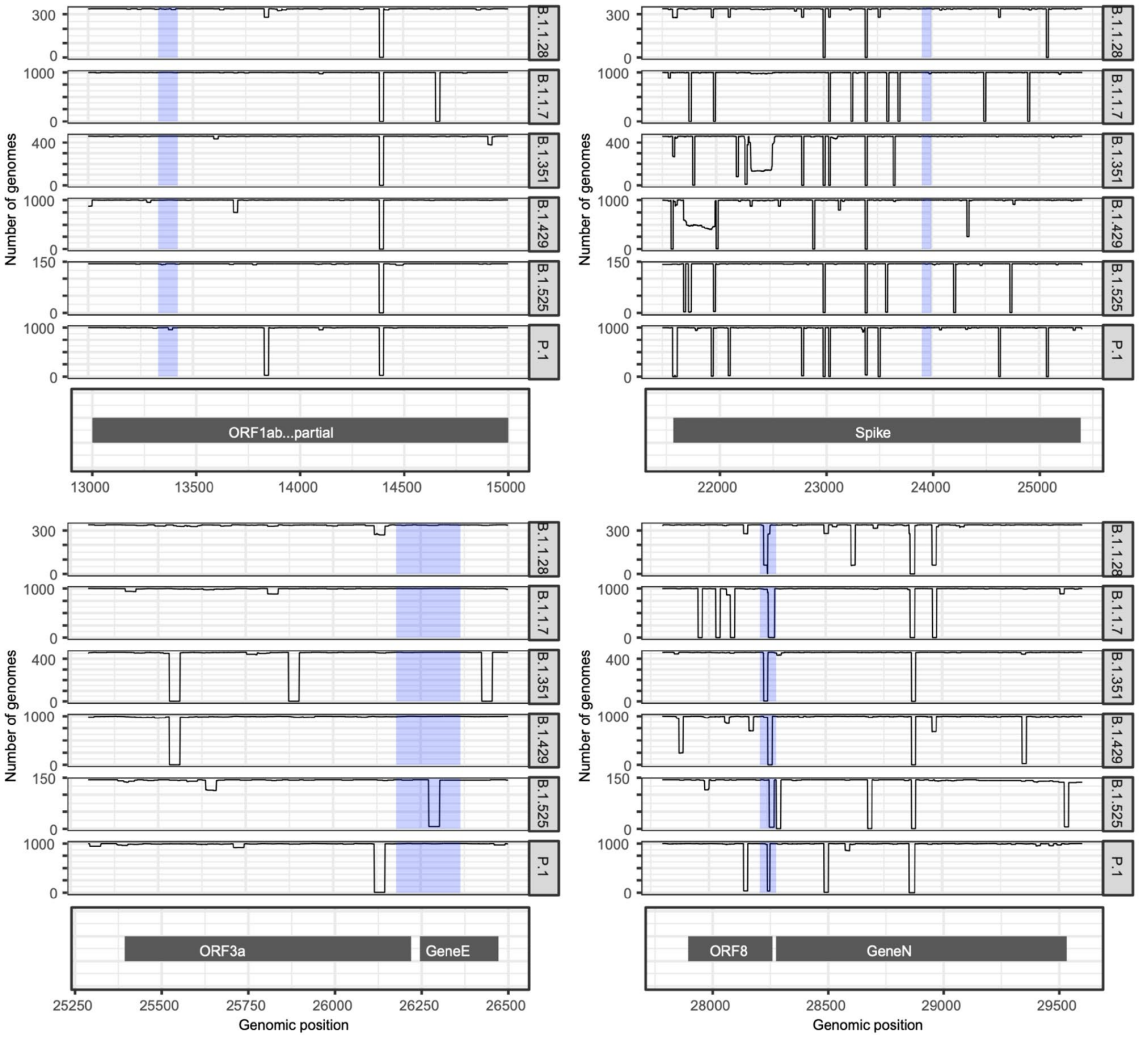
Number of SARS-CoV-2 population genomes that contain each reference kmer. Each plot shows each genome position at the X axis and the number of occurrences of the kmer starting at position X at the Y axis for each group of SARS-CoV-2 genomes. The shadowed blue regions represent the regions amplified by the PCR primers used in this study: upper left, primer NSP-3; upper right, primer S; lower left, primer E and lower right, primer N1.

We generated a population landscape for recently discovered SARS-CoV-2 lineages (P.1, B.1.1.7, B.1.351, B.1.1.28, B.1.429/B.1.427 or B.1.525). For each lineage, 1000 sequences (or all available sequences in some cases) from the GISAID repository were downloaded. The number of sequences per lineage as well as the country of origin is shown in Supplementary Table 4. The method used to generate the population landscape was the same as previously described, but the frequency of each reference kmer was obtained in each SARS-CoV-2 lineage (each variant was considered a different population). The exact sequences for both NSP-3 primers, both E primers, both S primers and the forward N1 primer were present in more than 99% of the SARS-CoV-2 genomic sequences, irrespective of the lineage. However, the reverse N1 primer had a distinctive behavior. Its exact sequence was present in more than 99% of the genomes of lineage P.1, B.1.351 and B.1.1.28, but it was not found in the B.1.429 genomes and was found in only 1 and 5 of the B.1.1.7 and B.1.525 genomes, respectively (out of 1000 and 144, respectively). This data indicates the presence of one point mutation in the region of the reverse N1 primer in some lineages which could interfere with the N1 amplicon generation (Fig. 4). Additionally, we generated conservation landscapes for the regions of the N2 oligos (CDC protocol) and the E and RdRp oligos from the Charité/Berlin (WHO) protocol. We found very high levels of conservation for almost all primer regions except for the reverse E primer which is found in only 4% of B.1.525 genomes (Supplementary Fig. 3).

The end-point PCR test was experimentally validated on 39, 37, 9 and 24 samples of SARS-CoV-2 samples identified by whole-genome sequencing as B.1.1.7, P1, B.1.429/B.1.427, and B.1.617.2/ AY respectively. The results showed specific bands corresponding to positive samples for 38 (out of 39), 37 (out of 37), 6 (out of 9) and 24 (out of 24) samples, respectively (Figure sup 4; Supplementary Table 3). The results showed no difference in the N1 amplicon generation between the different lineages suggesting that the point mutation found at the *in-silico* analysis is not interfering with the amplification.

Therefore, we propose that the population conservation landscape may be used as an epidemiological surveillance tool since such a landscape could be generated from the genomes of any newly discovered SARS-CoV-2 variant. This tool could be useful for the in silico analysis of PCR amplification efficiency and to design new PCR primers if required.

### End-point PCR is highly specific for the SARS-CoV-2 virus

**Figure 5:**
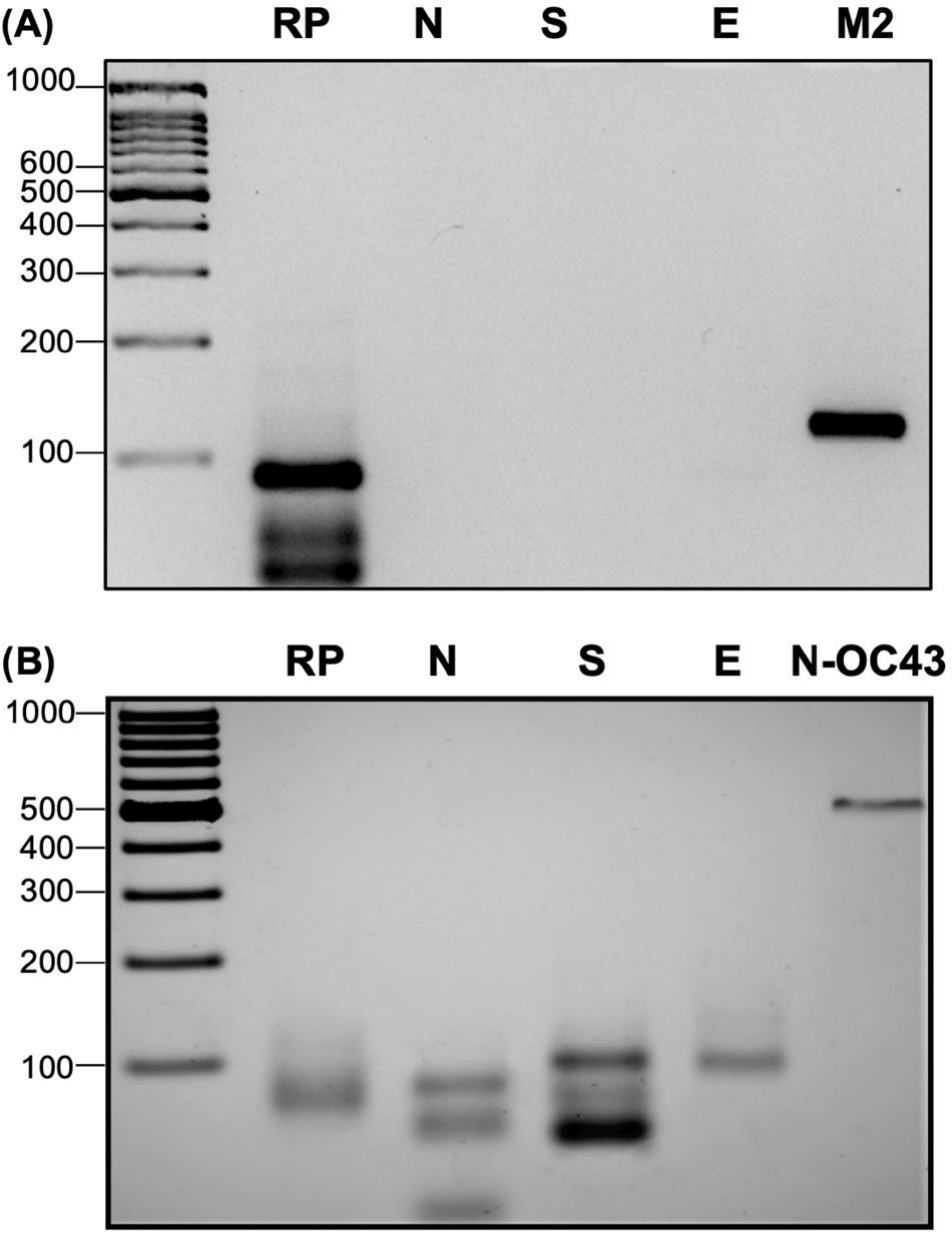
Assessment of the specificity of the SARS-CoV-2 detection test. (**A**) 4% agarose gel showing that the primers used for SARS-CoV-2 detection did not amplify H1N1 influenza virus RNA. (**B**) 4% agarose gel revealing that the test is adequate to discriminate SARS-CoV-2 from other coronaviruses such as β-coronavirus-OC43. M2, a specific fragment of ORF-7, and N1-OC43, a specific amplicon of the N gene, were used as positive controls for H1N1 influenza virus and β-coronavirus, respectively.

To determine whether this test can discern among other viruses that produce similar symptoms to those of moderate-intensity SARS-CoV-2 infection (Supplementary Table 7), we used RNA from influenza A H1N1 and HCoV-OC43. End-point PCR of these RNA samples showed no amplification fragments of the expected size (Fig. 5), indicating that this test is highly specific for the SARS-CoV-2 virus. This observation was also supported by the environmental conservation landscape, in which no kmers identical to any probe sequence were found (Fig. 2A). In addition, an in-silico analysis was done to generate PCR amplicons using either the Middle East respiratory syndrome-related coronavirus (MERS-CoV) genome (Accession: KU740200.1) or the SARS coronavirus HSR 1 (accession AY323977.2) and any of the following primers: primers S, E or NSP-3. No amplicons could be generated suggesting that these primers are specific for SARS-CoV-2 amplification.

## Discussion

The COVID-19 pandemic has evidenced large differences in the response capacity of health systems around the world. Thus, social distancing and epidemiological surveillance through diagnostic tests have become the best strategies to contain the virus by enabling decisions regarding mobility and isolation for positive cases and their close contacts. Unfortunately, developing countries with limited economic resources, where public health systems have been overwhelmed, have found it difficult to achieve a wide coverage of diagnostic tests for their population. In addition, the lack of adequate hospital infrastructure in rural communities located far from cities precludes the implementation of automated diagnostic tests such as RT-qPCR. Hence, economic disparities have prevented the general population from having access to diagnostic tests. Notably, since some governments have imposed few control measures for international travelers, such countries have been considered “touristic oases”, becoming major tourist destinations during the COVID-19 pandemic^25^, resulting in a latent risk as new pathogenic variants introduced by migratory and tourist flows could propagate in the local population. Therefore, the epidemiological surveillance picture around the world is complex.

In this work, we developed an accurate, low-cost diagnostic test based on end-point RT-qPCR that could be used as an alternative to classical RT-PCR diagnostics since minimal infrastructure is needed for this test. PCR is commonly used to characterize either the presence of specific bacteria or for pathogen detection. DNA signatures can specifically detect the presence of some specific organism or organisms (target genomes) without presenting cross-reactivity with other organisms (background genomes). Different pipelines and algorithms have been created to pinpoint DNA signatures. KPATH, Insignia and TOPSI are all devoted to finding DNA signatures^26–28^, and some of them use Primer3 software to calculate thermodynamic parameters and to design related sets of PCR assays. However, one important limitation common to all these high-throughput signature design programs is the requirement that any selected conserved sequence should be present in all target genomes; this criterion is difficult to meet for viral genomes due to their small size and their high mutation rates.

Lopez-Rincon et al.proposed a deep learning method to identify 21-bp sequences capable of discriminating SARS-CoV-2 from other coronaviruses. In this work, 10 features capable of classifying SARS-CoV-2 versus SARS-CoV, MERS-CoV and other coronaviruses were identified and further experimentally validated with one candidate primer set to show that their method could output successful PCR primers. However, these authors did not characterize the behavior of their approach in the presence of SARS-CoV-2 variants, as only 66 SARS-CoV-2 samples were used, which surely is an underrepresentation of the natural variability of the SARS-CoV-2 genome. In addition, the efficiency of their PCR was not addressed since the establishment of an alternative PCR assay was not the main goal of the work^29^.

As part of our work, we developed a PCR-design method based on a comprehensive conservation analysis of environmental sequences and SARS-CoV-2 population genomes. Our method is highly scalable and can be applied to any set of target and background sequences. Notably, it generates a straightforward representation of the conservation level and can be applied over sequences with high mutation rates.

Indeed, to develop a diagnostic test, it is important to consider the variability of the causative agent. Since SARS-CoV-2 was identified as the causative agent of COVID-19, it was soon demonstrated that although this virus possesses a low mutation rate (8.69 × 10^–4^ per site/year)^30^, it is constantly adapting. In addition, some of the changes in the viral genome affect its fitness, giving rise to new variants with higher pathogenicity than the original Wuhan lineage^31^. In an initial study, Wang et al*.* reported that even though mutations at the nucleotide and amino acid levels were relatively rare, some genome positions had high mutation rates (approximately 30%)^32^. Hence, several efforts have been made to design a wide variety of RT-PCR primers and probes to detect SARS-CoV-2. By May 2020, at least 19 different sets of RT-PCR assays had been proposed^33^.

The importance of genomic variants lies in their effect on the accuracy of diagnostic tests. For SARS-CoV-2, several studies have reported a possible decrease in test sensitivity due to their variants. Thus, some studies have monitored SARS-CoV-2 variation specifically in the regions targeted by RT-PCR primers and probes^34,35^. For example, the B1.1.7 strain from the United Kingdom accumulated 17 genomic mutations with respect to the Wuhan strain. One of these mutations involved a deletion in the 69/70 position of the S gene, which resulted in reduced diagnostic test sensitivity, as the S region is used as a target in several RT-qPCR diagnostic kits for SARS-CoV-2 detection^36^. These reports highlight the need for epidemiological surveillance as SARS-CoV-2 propagates around the world and mutates. Although one approach has been developed to identify variants exclusively in the primer/probe regions of existing PCR assays^37^, it is not designed to suggest regions for new primer sequences when existing PCR assays rely on hypervariable regions. The population conservation landscape generated in this work can also be used as a surveillance tool to inspect for common mutations in any given set of population genomes as new variants emerge. So, it can also be used to highlight new conserved regions for primer analysis and further experimental validation or as a diagnostic tool to test if current detection protocols rely on variable regions. Besides, it also could be applied to other pathogens to look for conserved regions as long as sequencing data is available; or as a tool to monitor genomic variability of agents causing upcoming pandemics. Although this tool can be used to reduce the number of candidate sequences to test for primer design, it does not reduce the resources required during the validation stage of any new protocol.

The primers used in the developed end-point PCR test are highly specific for SARS-CoV-2, being able to discriminate among other phylogenetically related coronaviruses (HCoV-OC43) that produce similar common cold symptoms (Table S4). Ct values obtained from RT-qPCR are semiquantitative measurements that can provide information about the course of SARS-CoV-2 infection (viral load and transmissibility). Although end-point PCR results cannot be represented as Ct values, the test developed in this study was robust enough to detect positive samples in patients who were diagnosed by RT-qPCR and whose Ct values were in the range of 16–34, indicating that its implementation should be relevant from the manifestation of the first symptoms until 18 days after the appearance of symptoms^38,39^. However, performing the test either within the first 7 days of virus incubation or in convalescent patients would be inefficient since the RT-qPCR measurements would exceed a Ct value of 35 with a viral load of 50–500 units/ml in both cases, which would require a lower detection limit than 7 particles per µl^40,41^. By other hand, endpoint PCR is a qualitative method that requires a validation by an agarose gel which is subject to human interpretation.

Although two tests were analyzed in this work, the test E showed higher sensitivity and was therefore selected for further analysis. Notably, when N1, Nsp3 and S primers were used in samples with Ct values greater than 30, diffuse nonspecific bands of genomic bulk were generated, complicating an accurate SARS-CoV-2 diagnosis. Therefore, a multiplex test was not considered. In contrast, in samples with higher Ct values (18–25), homogeneous bands of the expected size were obtained. This phenomenon might be due to the viral/genomic RNA concentration, where the primers will tend to specifically bind to their target regions in the virus genome at higher viral loads, whereas at low viral loads, they will nonspecifically bind genomic RNA. On the other hand, the pair of primers that bind to the 5´ region of the E gene also presented a 90 bp nonspecific band when either negative samples or samples with Ct values higher than 35 were used; however, given the expected size of the amplicon from the virus (185 bp), the nonspecific band did not interfere with the interpretation of the results, thus increasing test sensitivity (Table 2 , fig. sup 5 ).

Silva et al. described a method based on end-point PCR using primers directed to the 5´ end of the E gene^42^. Accordingly, this region is part of the fragment that is being amplified by our pair of E primers. Our results regarding the detection limit are in agreement. We observed that the 5´ end of the E gene possesses low variability, showing no variability in more than 34,000 SARS-CoV-2 genomes, including those of the new variants. Therefore, we propose that this region of the viral genome is an excellent candidate for the development of new diagnostic tests.

The confidence of a test is mainly determined by the number of false negative results. A false negative result was defined as a case in which a person with SARS-CoV-2 infection obtained a negative RT-qPCR result but tested positive in a subsequent test^43^. False negative results may occur due to low viral load, low test sensitivity, the site and quality of sampling, the stage of disease and degree of viral concentration or clearance, and to the prevalence of the disease in the population^44^. Remarkably, our end-point PCR evaluated only 2% of the samples with a previous RT-qPCR-positive result as negative^24^. Notably, these samples showed Ct values higher than 35, which was above the detection limit of our test. Therefore, the resulting sensitivity and specificity values suggest that this end-point PCR test would allow a diagnosis of true negative samples with a low margin of error, decreasing the likelihood of a false diagnosis.

## Conclusions

The specific end-point PCR diagnostic test developed in this work represents a low-cost alternative to the gold-standard qRT-PCR diagnostic. Moreover, the bioinformatic approach developed in this work can be applied to guide the design of PCR primers based on the conservation of reference kmers across any set of target genomes. Since it can be easily applied over thousands of viral genomes to compare highly divergent strains, this method could be used to monitor population sample variability as new SARS-CoV-2 variants emerge.

## Supplementary Information

Supplementary Information 1.

Supplementary Information 2.

Supplementary Information 3.

## Data Availability

The pipeline can be downloaded from https://github.com/INMEGEN/conservationLandscape.
