# Supplementary material for: End-point RT-PCR based on a conservation landscape for SARS-COV-2 detection
Source: Sci Rep. 2022 Mar 19;12:4759. doi: 10.1038/s41598-022-07756-6 (PMC8933765; doi:10.1038/s41598-022-07756-6)
Supplement: Supplementary file 1 — Supplementary Information 1. [file 41598_2022_7756_MOESM1_ESM.docx]

**END-POINT RT-PCR BASED ON A CONSERVATION LANDSCAPE FOR SARS-COV-2 DETECTION**

**SUPPLEMENTARY INFORMATION**

**SUPPLEMENTARY TABLES**

| **Organism** | **Number of sequences** | **Accesion list ( if N < 10)** |
| --- | --- | --- |
| Bocavirus | 24 | NA |
| Chlamydophila pneumoniae | 6 | AE001363.1, AE002161.1, AE009440.1, BA000008.3, CP001713.1, CP001714.1 |
| Coxsackievirus | 52 | NA |
| Haemophilus influenzae | 74 | NA |
| Human adenovirus | 1 | MN513338.1 |
| Human coronavirus NL63 | 1 | AY567487.2 |
| Human coronavirus 229E | 1 | AF304460.1 |
| Human coronavirus OC43 | 1 | AY585228.1 |
| Human metapneumovirus | 5 | AF371337.2, AY297749.1, AY525843.1, FJ168778.1, FJ168779.1 |
| Human respiratory syncytial virus B | 26 | NA |
| Influenza A virus | 1104 | NA |
| Influenza B virus | 8 | AF101982.1, AF102017.1, J02094.1, J02095.1, J02096.1, K00423.1, K01395.1, M14880.1 |
| Legionella pneumophila | 130 | NA |
| Mycoplasma pneumoniae | 81 | NA |
| Parainfluenza virus | 2 | AB543336.1, KT215610.1 |
| Rhinovirus | 165 | NA |
| Streptococcus pyogenes | 229 | NA |

**SUPPLEMENTARY TABLE 1.** Number of sequences analyzed per organism. All available sequences in the European Nucleotide Archive (ENA) (at the time of this analysis) were downloaded.

| **Nucleotide substitution or lineage** | **Number of sequences** |
| --- | --- |
| B.1.1.28 | 339 |
| B.1.429/B.1.427 | 1000 |
| B.1.1.7 | 1000 |
| B.1.351 | 460 |
| B.1.525 | 144 |
| P.1 | 1000 |
| N_G204R | 1000 |
| N_I292T | 1000 |
| N_P13L | 1000 |
| N_R203K | 1000 |
| N_S194L | 1000 |
| NS3_G251V | 1000 |
| NS3_Q57H | 1000 |
| NS3_V13L | 1000 |
| NS8_L84S | 1000 |
| NS8_Q27stop | 1000 |
| NS8_S24L | 1000 |
| NSP12_A97V | 1000 |
| NSP12_P323L | 1000 |
| NSP13_P504L | 1000 |
| NSP13_Y541C | 1000 |
| NSP2_D268del | 1000 |
| NSP2_I559V | 1000 |
| NSP2_P585S | 1000 |
| NSP2_T85I | 1000 |
| NSP3_T1198K | 1000 |
| NSP5_G15S | 1000 |
| NSP6_L37F | 1000 |
| Spike_A222V | 1000 |
| Spike_D614G | 1000 |
| Spike_E484K | 228 |
| Spike_E484Q | 52 |
| Spike_E780Q | 481 |
| Spike_G476S | 35 |
| Spike_H69del | 1000 |
| Spike_K417N | 479 |
| Spike_L18F | 802 |
| Spike_N439K | 1000 |
| Spike_N501Y | 1000 |
| Spike_S477 | 1000 |
| Spike_T20N | 61 |
| Spike_T478I | 179 |
| Spike_V1176F | 1000 |
| Spike_V483A | 60 |
| Spike_Y145del | 385 |
| Spike_Y453F | 153 |
| **SUBTOTALS** | |
| Variant-specific sequences | 3,943 |
| Other sequences | 31,915 |

**SUPPLEMENTARY TABLE 2.** Number of SARS-CoV-2 genomic sequences downloaded from the GISAID database per nucleotide substitution or lineage. A maximum of 1000 sequences was downloaded in each case. If there were fewer than 1000 sequences reported in the database, then all available sequences were downloaded.

| **Name** | **Sequence** |
| --- | --- |
| N-OC43- F | 5' TGCCTATTGCACCAGGAGTC 3' |
| N-OC43- R | 5' TCAGCCATGTCAGGTGTTAC 3' |
| InfA-F | 5' GACCRATCCTGTCACCTCTGAC 3' |
| InfA-R | 5' AGGGCATTYTGGACAAAKCGTCTA 3' |

**SUPPLEMENTARY TABLE 4.** Primer sequences for the detection of the HcoV-OC43 and H1N1 viruses.

|  |  |  |  |  |
| --- | --- | --- | --- | --- |
| **RP** | **N1** | **S** | **E** | **DIAGNOSTIC** |
| ✓ | ✓ | ✓ | ✓ | POSITIVE |
| ✓ | X | ✓ | ✓ | POSITIVE |
| ✓ | X | X | ✓ | POSITIVE |
| ✓ | ✓ | X | ✓ | POSITIVE |
| NP | NP | NP | NP | INCONCLUSIVE |
| ✓ | X | X | X | NEGATIVE |

**SUPPLEMENTARY TABLE 5. Classification criteria for diagnosis of SARS-CoV-2 by endpoint PCR (TestE**). X = A marker is considered as absent even in the presence of unspecific bands shorter than 100 pb. ✓= good definition and enriched amplification products.

NP= does not present bands

| **Origin** | **Number of sequences** | **Lineage** |
| --- | --- | --- |
| United Kingdom | 1000 | B.1.1.7 |
| Brazil | 1000 | P.1 |
| South Africa | 460 | B.1.351 |
| Brazil | 339 | B.1.1.28 |
| California | 1000 | B.1.429/B.1.427 |
| United Kingdom | 144 | B.1.525 |

#### SUPPLEMENTARY TABLE 6. Number of sequences downloaded from GISAID and origin for each SARS-CoV 2 lineage

| **Symptomatology** | **SARS-CoV-2** | **OC43** | **H1N1** |
| --- | --- | --- | --- |
| Fever or chills | ✓ | ✓ | ✓ |
| Cough | ✓ | ✓ | ✓ |
| *Shortness of breath or difficulty breathing | ✓ |  |  |
| Fatigue | ✓ |  | ✓ |
| Body aches | ✓ | ✓ | ✓ |
| Headache | ✓ | ✓ | ✓ |
| loss of taste or smell | ✓ |  |  |
| Sore throat | ✓ | ✓ | ✓ |
| Nasal congestion | ✓ | ✓ | ✓ |
| Diarrhea, vomit | ✓ | ✓ |  |

**SUPPLEMENTARY  TABLE 7.** Symptomatology for SARS-CoV-2 infection and other respiratory infections

**SUPPLEMENTARY FIGURES**


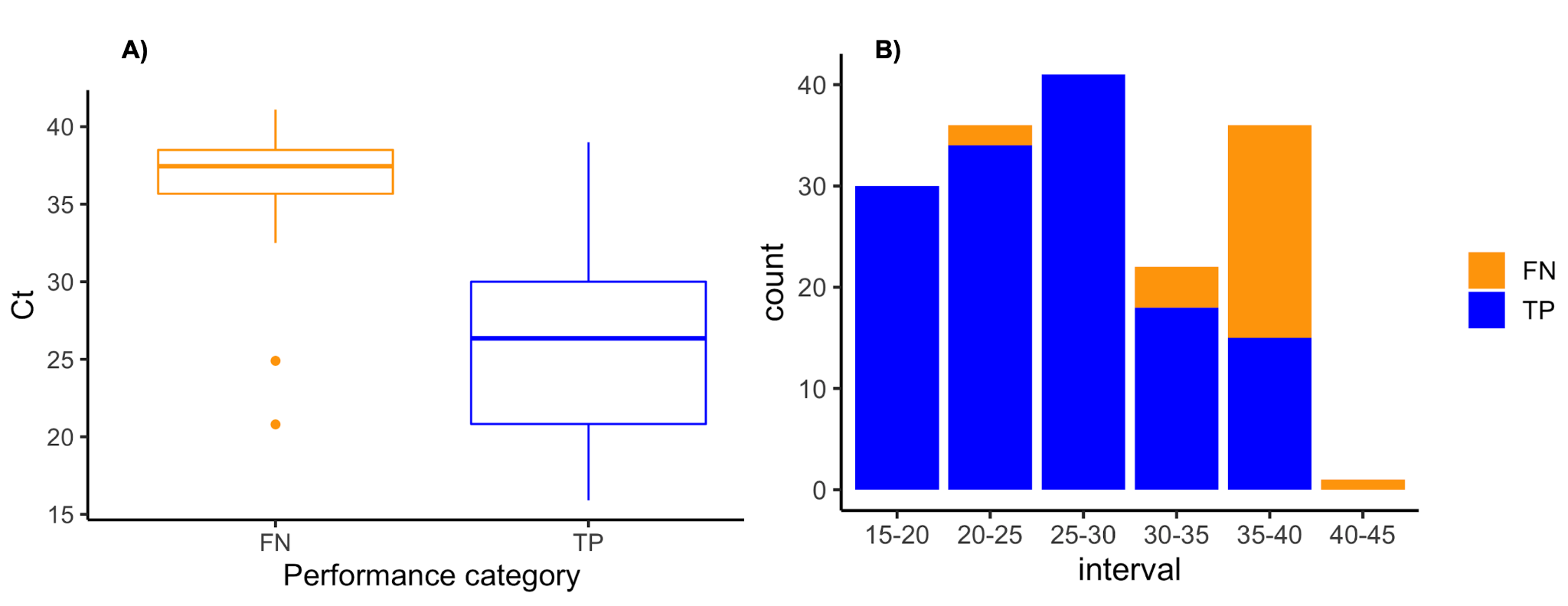


**SUPPLEMENTARY FIGURE 1. NUMBER OF TRUE POSITIVES AND FALSE NEGATIVES FOR INCREASING RANGES OF CT VALUES**. A) Ct distribution for False Negative and True Positive samples. B) Number of True Positives and False Negatives for different Ct ranges. True Positives are represented in blue, False negatives are represented in orange.

**
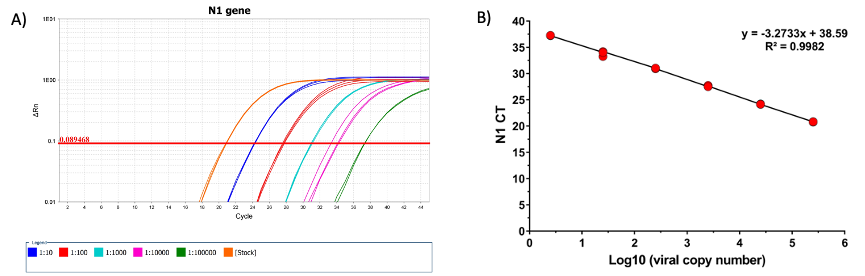
**

#### SUPPLEMENTARY FIGURE 2. STANDARD CURVE FOR GENE N. A) Real-time PCR amplification curves of viral dilutions for the N gene. Each point of the standard curve corresponds to three experimental replicates. B) Curve obtained from serial dilutions of previously RT-qPCR-positive control samples.


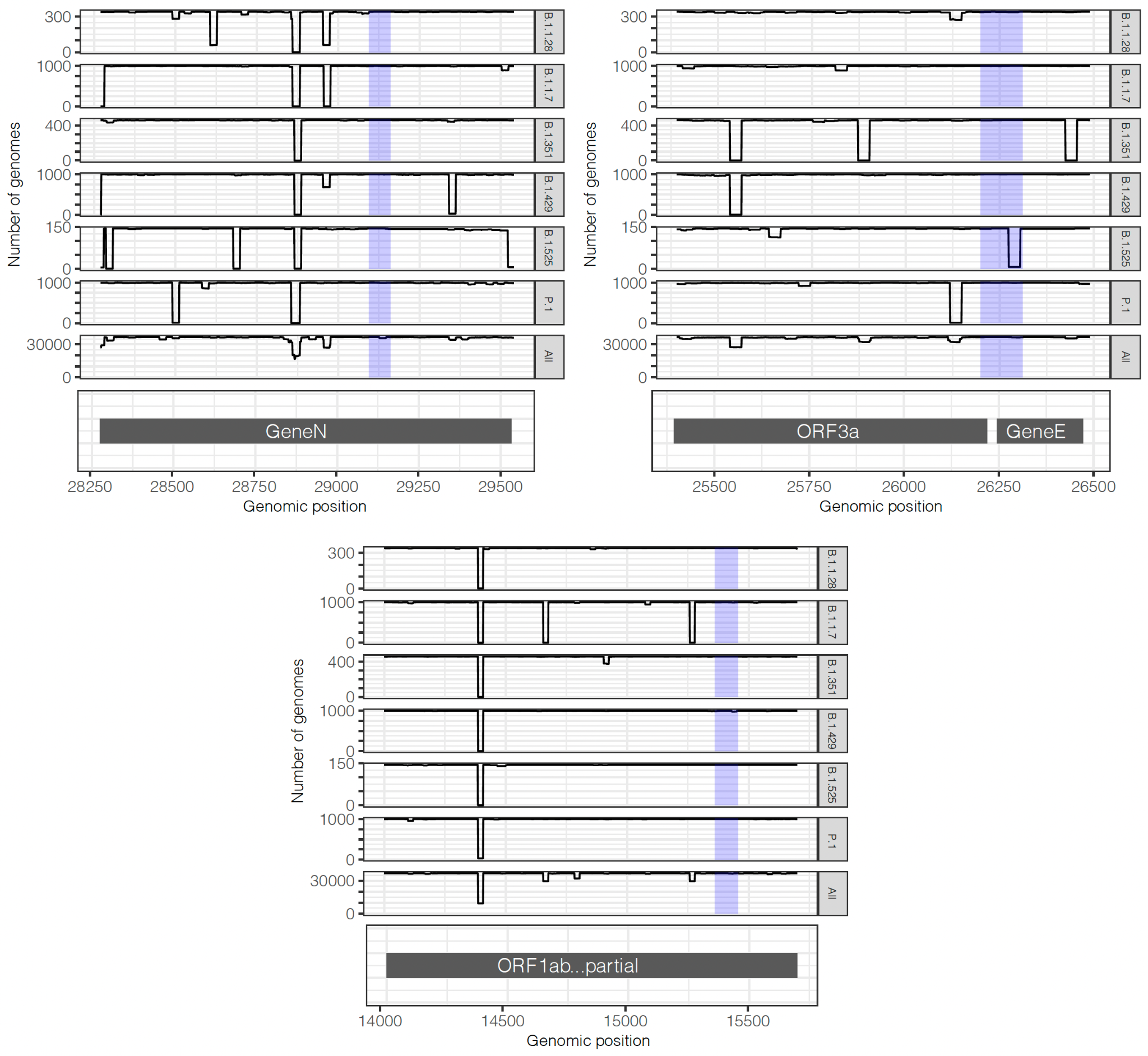


**SUPPLEMENTARY FIGURE 3. Number of SARS-CoV-2 population genomes that contain each reference kmer for primers of different SARS-CoV-2 identification protocols**. Each plot shows each genome position at the X axis and the number of occurrences of the kmer starting at position X at the Y axis for each group of SARS-CoV-2 genomes. The shadowed blue regions represent the regions amplified by the PCR primers probes used identified in this study: upper left, primer N2 CDC; upper right, primer E Charité Berlín; lower, primer RdRp Charité Berlín.


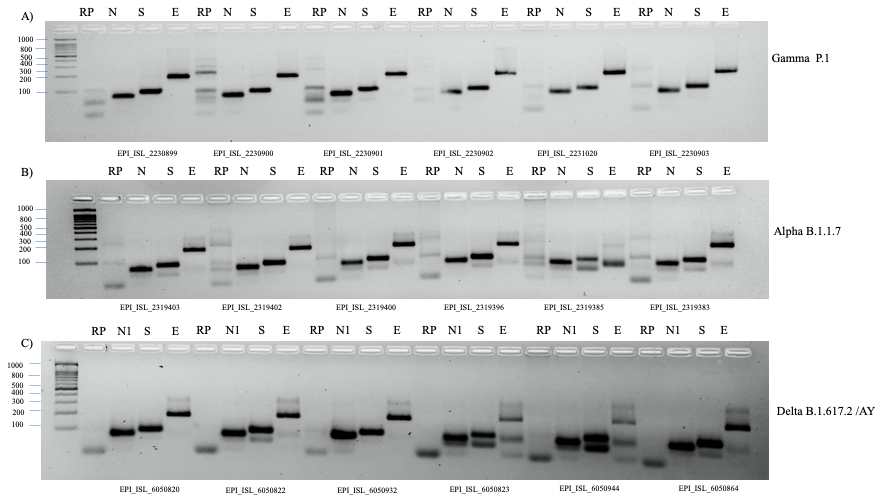


**SUPPLEMENTARY FIGURE 4. End-point RT-PCR evaluation on positive samples of some SARS-CoV-2 variants of concern.** Panel (A) shows a representative agarose gel with samples carrying the gamma variant (P.1). In panel (B) representative agarose gel of samples carrying the alpha variant (B.1.1.7) is shown. Panel (C) displays samples carrying the delta variant (B.1.617). GISAID accession number of each sample is provided at the bottom of each image.


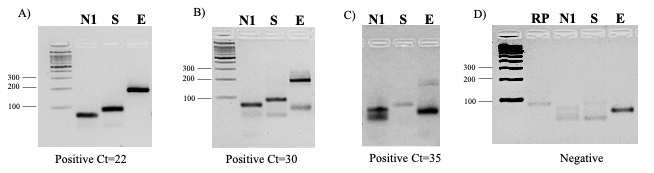


**SUPPLEMENTARY FIGURE 5. Comparison of RT-PCR amplification products in samples with distinct Cts values.** A) 4% agarose gel showing the RT-PCR amplification products from A) a sample with a Ct value of 21, B) a sample with a Ct value of 30, C) sample with Ct = 35, and D) a negative sample for the N1, S and E primers. To note, when N1, S and E primers are used in positive samples with higher viral titers, enriched amplification products are obtained; hence, higher RNA viral loads lead to a better definition in the RT-PCR amplification products. In contrast, in negative samples, unspecific bands shorter than 100 pb are observed.
